# Diagnostic Utility of Trio–Exome Sequencing for Children With Neurodevelopmental Disorders

**DOI:** 10.1001/jamanetworkopen.2025.1807

**Published:** 2025-03-25

**Authors:** Xiaoping Lan, Xiaojun Tang, Wenhao Weng, Wuhen Xu, Xiaozhen Song, Yongchen Yang, Hong Sun, Haiyun Ye, Hong Zhang, Guangjun Yu, Shengnan Wu

**Affiliations:** 1 Department of Clinical Laboratory, Shanghai Children’s Hospital, School of Medicine, Shanghai Jiao Tong University, Shanghai, China; 2 Institute of Pediatric Infection, Immunity, and Critical Care Medicine, Shanghai Jiao Tong University School of Medicine, Shanghai, China; 3 College of Health Science and Technology, Shanghai Jiao Tong University School of Medicine, Shanghai, China; 4 Shanghai Engineering Research Center for Big Data in Pediatric Precision Medicine, Shanghai Children’s Hospital, School of Medicine, Shanghai Jiao Tong University, Shanghai, China; 5 Center for Biomedical Informatics, Shanghai Children’s Hospital, School of Medicine, Shanghai Jiao Tong University, Shanghai, China; 6 Department of Ophthalmology, Shanghai Children’s Hospital, School of Medicine, Shanghai Jiao Tong University, Shanghai, China; 7 Shanghai Children’s Hospital, School of Medicine, Shanghai Jiao Tong University, Shanghai, China; 8 Shenzhen Maternity and Child Healthcare Hospital, Southern Medical University, Shenzhen, Guangdong Province, China

## Abstract

**Importance:**

Copy number variants (CNVs) and single-nucleotide variations (SNVs) or insertions and deletions are key genetic contributors to neurodevelopmental disorders (NDDs). Traditionally, chromosome microarray and exome sequencing (ES) have been used to detect CNVs and single gene variants, respectively.

**Objective:**

To identify genetic variants causing NDDs and evaluate the diagnostic yield and clinical utility of ES by simultaneously analyzing CNVs and SNVs in patients with NDDs and their biologic parents (trios).

**Design, Setting, and Participants:**

This retrospective cohort study included pediatric patients with suspected NDDs who visited Shanghai Children’s Hospital between January 1, 2018, and December 31, 2023. ES was used to investigate trios (trio-ES) including patients with NDDs who remained undiagnosed after phenotype identification and underwent gene panel testing, multiplex ligation-dependent probe amplification, or karyotyping. Comprehensive clinical and laboratory data were collected. Data were analyzed from July 2022 to December 2023.

**Exposure:**

NDDs, characterized by global developmental delay or intellectual disability.

**Main Outcomes and Measures:**

The study measured the overall diagnostic yield of SNVs and CNVs in the NDD cohort as well as within NDD syndromic subtypes.

**Results:**

Of the 1106 patients with NDDs, 731 (66.1%) were male. The mean (SD) age of patients at diagnosis was 3.80 (2.82) years. The overall diagnostic yield of trio-ES was 46.1% (510 diagnoses among 1106 patients), with 149 CNVs (13.5%), 355 SNVs (32.1%), and 4 cases of uniparental disomy (0.4%). Codiagnosis of SNVs and CNVs occurred in 2 cases (0.2%). Among the trios, 812 candidate germline variants were identified, including 634 SNVs (78.1%), 174 CNVs (21.4%), and 4 cases of uniparental disomy (0.5%). Of these, 423 SNVs (66.7%) and 157 CNVs (90.2%) were diagnostic variants, while 211 SNVs (33.3%) and 17 CNVs (9.8%) were variants of uncertain significance. Sixteen CNVs smaller than 20 kilobase were detected using ES.

**Conclusions and Relevance:**

In this cohort study, trio-ES, by simultaneously detecting SNVs and CNVs, achieved a diagnostic yield of 46.1%. Trio-ES may be particularly applicable for identifying small CNVs and recessive genetic diseases involving both SNVs and CNVs. These findings suggest that in clinical practice, simultaneously analyzing SNVs and CNVs using trio-ES data has a favorable genetic diagnostic yield for children with NDDs.

## Key Points

**Question:**

What is the diagnostic yield of the simultaneous analysis of single-nucleotide variations (SNVs) and copy number variants (CNVs) using exome sequencing for genetic testing of patients with neurodevelopmental disorders (NDDs) and their biologic parents (trio-ES)?

**Findings:**

This cohort study of 1106 pediatric patients with NDDs identified 580 diagnostic variants: 423 SNVs and 157 CNVs. The overall diagnostic yield of trio-ES was 46.1%, with 13.5% for CNVs, 32.1% for SNVs, and 0.4% for uniparental disomy.

**Meaning:**

These findings suggest that using trio-ES data for the simultaneous detection of CNVs and SNVs can achieve a favorable diagnostic yield in pediatric patients with NDDs.

## Teaser

This cohort study examines the diagnostic yield and clinical utility of using exome sequencing to analyze single nucleotide variants and copy number variants simultaneously in genetic testing for children with neurodevelopmental disorders.

## Introduction

Neurodevelopmental disorders (NDDs) encompass a range of conditions resulting from impaired brain and nervous system development, often leading to cognition, language, behavior, and motor skill delays emerging in early childhood. NDDs include global developmental delay and intellectual disability (GDD-ID), autism spectrum disorders (ASD), attention-deficit/hyperactivity disorder (ADHD), communication disorders, and motor disorders, which frequently co-occur with congenital anomalies (CAs) and epilepsy or infantile spasms (EIS).^1,2^ Among these, GDD-ID is a key feature of NDDs, affecting 1% to 3% of the general population,^3,4^ with the term *ID* referring to individuals aged 5 years or older and *GDD* referring to individuals younger than 5 years.^5^ The etiology of NDDs primarily involves environmental and genetic factors, with genetic causes being the most prevalent, accounting for 25% to 50% of cases.^6^

Genetic testing plays a crucial role in providing clinical and etiological diagnoses for patients and families, yet there is currently no single genetic assay capable of detecting the different types of genomic aberrations in NDDs simultaneously.^4^ Typically, chromosomal microarray (CMA) is used to identify copy number variants (CNVs) and uniparental disomy (UPD), while exome sequencing (ES) targets single gene variants like single-nucleotide variations (SNVs) and small insertions or deletions.^7,8,9,10,11^ For undiagnosed patients, further tests, such as fragile X testing or whole-genome sequencing, are performed according to the clinical features.^12,13^ Using ES data for scrutinizing structural variants, such as CNVs and UPD, presents a cost-effective strategy to enhance the yield of genetic diagnosis. Recent advancements in bioinformatics have allowed CNV identification using ES data,^14,15^ improving clinical application in genetic disorders.^16,17^ Published studies have indicated that simultaneous analysis of SNVs and CNVs using ES or clinical ES data in cohorts of patients with NDDs can significantly increase diagnostic yields, making it a recommended first-tier genetic testing approach.^18,19,20,21^ Nevertheless, to our knowledge, the diagnostic yield and clinical suitability of ES for detecting SNVs and CNVs simultaneously in patients with NDDs have not been systematically evaluated. In this study, we aimed to identify genetic variants causing NDDs and to evaluate the diagnostic yield and clinical utility of ES by simultaneously analyzing CNVs and SNVs in patients with NDDs and their biologic parents.

## Methods

### Study Design and Participants

The present cohort study was approved by the ethics committee of the Shanghai Children’s Hospital, School of Medicine, Shanghai Jiao Tong University, and written informed consent was signed by each participant or their legal representative. The study followed the Strengthening the Reporting of Observational Studies in Epidemiology (STROBE) reporting guideline for cohort studies.

**Figure 1.**
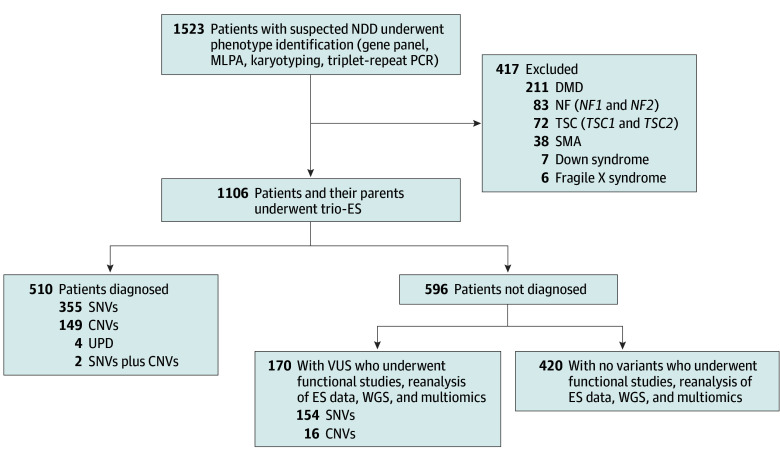
Flowchart of Study Design CNV indicates copy number variant; DMD, Duchenne muscular dystrophy; ES, exome sequencing; MLPA, multiplex ligation-dependent probe amplification; NDD, neurodevelopmental disorder; NF, neurofibromatosis; PCR, polymerase chain reaction; SMA, spinal muscular atrophy; SNV, single-nucleotide variation; trio-ES, trio-exome sequencing; TSC, tuberous sclerosis; UPD, uniparental disomy; VUS, variant of unknown significance; WGS, whole-genome sequencing.

This retrospective study initially included consecutive patients with suspected NDDs who visited Shanghai Children’s Hospital from January 1, 2018, to December 31, 2023. Patients with tuberous sclerosis (involving genes *TSC1* and *TSC2*), neurofibromatosis (involving genes *NF1* and *NF2*), Duchenne muscular dystrophy (involving gene *DMD*), spinal muscular atrophy (involving genes *SMN1* and *SMN2*), Down syndrome, and fragile X syndrome were excluded by precise phenotype identification and gene panel, multiplex ligation-dependent probe amplification (MLPA), karyotyping testing, and triplet-repeat primed polymerase chain reaction (PCR). The remaining patients with NDDs and their biologic parents, all of whom underwent ES, were included in the study cohort. Given the absence of biases in self- or parent-reported gender, age, or family history, the cohort offered a representative sample mirroring real clinical scenarios. The clinical manifestations, imaging data, and laboratory results were extracted from the medical records documented by well-trained pediatric neurologists. The flowchart of the study design from enrollment to genetic testing is shown in Figure 1.

### Outcomes

In this case-only cohort study, NDD was defined as the presence of a clinical phenotype of GDD-ID. The diagnosis of GDD-ID is made by meeting the following criteria: (1) abnormalities in gross or fine motor skills, speech or language, and cognition; (2) abnormalities in social or personal behavioral and adaptive functioning; and (3) deficits in perceptual and quantitative reasoning, verbal comprehension, abstract thought, comprehension of instructions and rules, memory, problem-solving, and learning from experience.^22,23^

### Exome Sequencing, Variant Interpretation, and Classification

We provide a detailed description of the exome sequencing and data analysis in the eMethods in Supplement 1. The interpretation and classification of variants are also described in detail in the eMethods in Supplement 1.

### Statistical Analysis

Involved diagnostic yield was calculated as the percentage of patients with positive results among patients with NDD in each group. Differences in the frequency between groups were investigated using χ^2^ test for categorical variables. Statistical analyses were performed and plots were created from July 2022 to December 2023 using GraphPad Prism, version 9.5.0 for Windows (GraphPad Software). Two-sided *P* < .05 was considered to show statistical significance.

## Results

### Cohort Information

Of 1523 consecutive pediatric patients with NDDs who visited our hospital, 417 with tuberous sclerosis, neurofibromatosis, Duchenne muscular dystrophy, spinal muscular atrophy, Down syndrome, or fragile X syndrome were excluded, and the remaining 1106 patients, along with their parents, were enrolled (N = 3318). Among these patients, 673 with GDD-ID (60.8%) had comorbidities, such as CAs, ASD, EIS, ADHD, and brain malformations (abnormal brain magnetic resonance imaging [MRI] findings), and were classified as having a syndromic type. The remaining 433 patients with only GDD-ID (39.2%) were classified as having a nonsyndromic type (Table). The next-generation sequencing strategy of trio-ES was used for all participants. All the patients were born to nonconsanguineous parents. Among the patients, 375 (33.9%) were female and 731 (66.1%) were male. The mean (SD) age of patients at diagnosis was 3.80 (2.82) years (range, 0-17 years). Most patients (851 [76.9%]) received trio-ES tests before age 5 years. Within the syndromic group, the same patient could have multiple comorbidities associated with GDD-ID, encompassing various subtypes of comorbidities. Among the 673 syndromic patients, 232 (34.5%) had abnormal brain MRI findings, and CAs were the most common comorbidity (212 patients [31.5%]), followed by ASD (147 [21.8%]), EIS (138 [20.5%]), and ADHD (110 [16.3%]). The detailed demographic and clinical information is given in the Table.

**Table.** Demographic and Clinical Characteristics and Diagnostic Yield of Patients With NDDs

Characteristic	Patients, No./total No. (%)					
	All	Diagnostic yield				
		SNV	CNV	SNV plus CNV	UPD	Total
Gender						
Female	375/1106 (33.9)	146/375 (38.9)	56/375 (14.9)	1/375 (0.3)	1/375 (0.3)	204/375 (54.4)
Male	731/1106 (66.1)	209/731 (28.6)	93/731 (12.7)	1/731 (0.1)	3/731 (0.4)	306/731 (41.9)
Subtype of syndromic NDD						
Abnormal MRI findings	232/673 (34.5)	89/232 (38.4)	30/232 (12.9)	0	0	119/232 (51.3)
Congenital anomalies	212/673 (31.5)	86/212 (40.6)	43/212 (20.3)	1/212 (0.5)	3/212 (1.4)	133/212 (62.7)
ASD	147/673 (21.8)	28/147 (19.0)	6/147 (4.1)	0	0	34/147 (23.1)
Epilepsy or infantile spasms	138/673 (20.5)	57/138 (41.3)	17/138 (12.3)	0	0	74/138 (53.6)
ADHD	110/673 (16.3)	28/110 (25.5)	14/110 (12.7)	0	0	42/110 (38.2)

Abbreviations: ADHD, attention-deficit/hyperactivity disorder; ASD, autism spectrum disorders; CNV, copy number variant; MRI, magnetic resonance imaging; NDD, neurodevelopmental disorder; SNV, single-nucleotide variation; UPD, uniparental disomy.

### Diagnostic Yields in the Cohort

**Figure 2.**
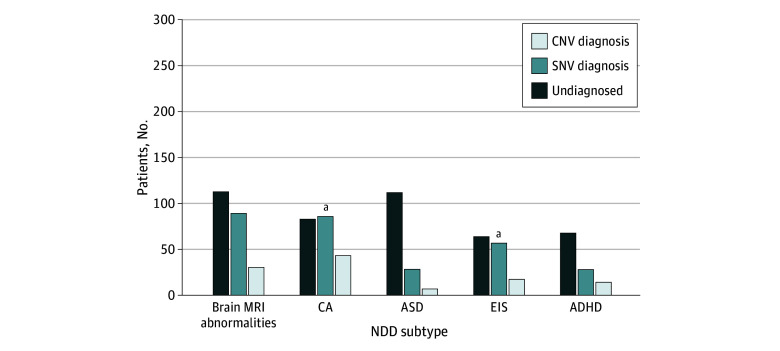
Diagnostic Yields for Different Subtypes of Syndromic Neurodevelopmental Disorders (NDDs) ADHD indicates attention-deficit/hyperactivity disorder; ASD, autism spectrum disorders; CA, congenital anomaly; CNV, copy number variant; EIS, epilepsy or infantile spasms; MRI, magnetic resonance imaging; SNVs, single-nucleotide variations. ^a^*P* < .05.

Simultaneous identification of SNVs and structure variants from trio-ES data resulted in an overall diagnostic yield of 46.1% (510 diagnoses among 1106 patients). Of these, CNVs constituted 149 of the diagnoses (13.5%), while SNVs constituted 355 (32.1%). Notably, there were 2 cases (patients P0299 and P0927) of recessive genetic diseases diagnosed by both SNV and CNV (eFigure in Supplement 1) and 4 cases of UPD (eTable 3 in Supplement 2), accounting for 0.2% and 0.4% of the total diagnostic yield, respectively. Within the genetically diagnosed patient cohort, CNVs accounted for 149 of the 504 diagnoses (29.6%) and SNVs accounted for 355 (70.4%). Among the 506 cases diagnosed by SNVs or CNVs, 401 cases (79.2%) were diagnosed as de novo variants (DNVs). Furthermore, among the 355 patients diagnosed with SNVs, 267 cases (75.2%) were DNVs. The overall diagnostic yield of de novo SNVs in this study was 24.1% (267 of 1106). The diagnostic yields of the different subgroups were 201 of 433 (46.4%) for nonsyndromic GDD-ID and 309 of 673 (45.9%) for syndromic GDD-ID, with no significant difference between them (*P* = .90). Among the 5 subtypes of syndromic GDD-ID, the diagnostic yield varied significantly, ranging from 34 of 147 (23.1%) for ASD to 133 of 212 (62.7%) for CAs. The diagnostic yields of the subtype with ASD were significantly lower compared with the subtype with CAs, EIS, abnormal brain MRI findings, and ADHD. However, the diagnostic yields of abnormal brain MRI findings, CAs, and EIS subtypes were not significantly different from each other (Table). The detailed numbers and diagnostic yields for SNVs and CNVs in all subtypes are provided in Figure 2.

### Spectrum of Candidate Variants

**Figure 3.**
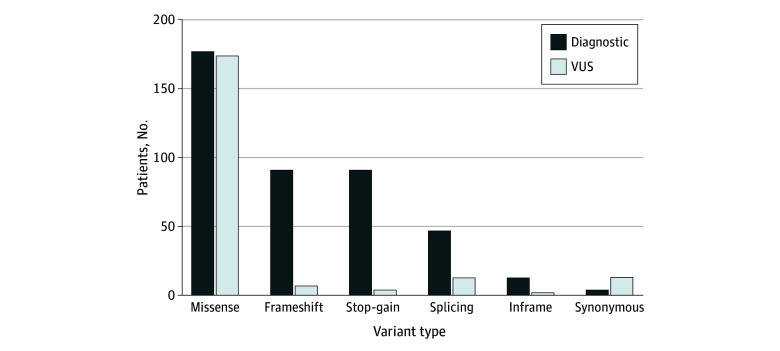
Spectrum of Single-Nucleotide Variations in the Cohort With Neurodevelopmental Disorders VUS indicates variant of unknown significance.

**Figure 4.**
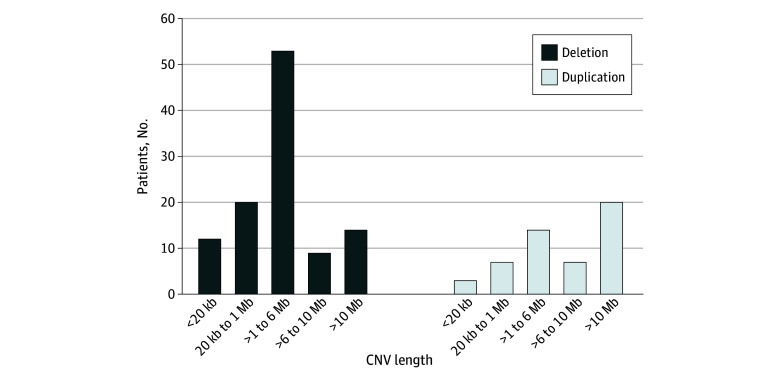
Spectrum of Diagnostic Copy Number Variants (CNVs) in the Cohort With Neurodevelopmental Disorders kb Indicates kilobase; Mb, megabase.

Through the analysis of the ES data of 1106 patients with NDDs, a total of 812 candidate germline variants were identified in 680 individuals (61.5%), including 634 SNVs (78.1%) (eTable 1 in Supplement 2), 174 CNVs (21.4%) (eTable 2 in Supplement 2), and 4 cases of UPD (0.5%) (eTable 3 in Supplement 2). Among the 634 SNVs, 423 (66.7%) were diagnostic variants (including pathogenic and likely pathogenic) (Figure 3) and 211 (33.3%) were variants of uncertain significance (Figure 3 and eTable 4 in Supplement 2). Among the 174 CNVs, 157 (90.2%) were diagnostic variants (including pathogenic and likely pathogenic) (Figure 4) and 17 (9.8%) were variants of uncertain significance.

### Spectrums of Diagnostic CNVs

A total of 157 diagnostic CNVs were identified in 151 (13.7%) of the individuals with NDDs, of which 108 (66.8%) were deletion variants (including 3 hemizygous or homozygous [P0957] deletions [2.8%] and 105 heterozygous deletions [97.2%]) and 49 (31.2%) were duplication variants. Additionally, out of the 155 disease-causing CNVs, 141 (91.0%) were identified as DNVs. The size of the 157 CNVs ranged from a single exon (124 base pair [bp]) (patient P0957) to an entire chromosome (155 megabase [Mb]) (patients P0287, P0428, and P1031). Among these CNVs, 115 (73.2%) were larger than 1 Mb, while 42 (26.8%) were smaller than 1 Mb. Within the subset of 106 CNVs smaller than 6 Mb, 84 (79.2%) were deletion variants, whereas only 22 (20.8%) were duplication variants. Fifty-one CNVs (32.5%) were larger than 6 Mb, including 27 duplication variants (52.9%) and 24 deletion variants (47.1%) (Figure 4). In the CNVs smaller than 6 Mb, a notably higher proportion of diagnostic variants were deletions (84 of 106 [79.2%]), whereas in CNVs larger than 6 Mb, duplications were more prevalent (27 of 51 [52.9%]). Notably, the largest duplication variants encompassed the entire X chromosome and were present in 2 of 151 patients with karyotype 47,XXY (1.3%) and 1 of 151 patients with 49,XXXXY (0.7%). Among 157 diagnostic CNVs, 19 (12.1%) were less than 100 kb, affecting only a limited number of exons within a single gene (including 16 deletions and 3 duplications) (eTable 2 in Supplement 2). Furthermore, 16 CNVs (10.2%) were smaller than 20 kilobase (kb) (Figure 4). Fourteen patients (87.5%) were diagnosed with exon-level CNV within a single gene, and 2 (12.5%) were codiagnosed with both SNVs and CNVs. CNVs smaller than 100 kb and 20 kb had diagnostic yields of 1.7% (19 of 1106) and 1.4% (16 of 1106), respectively, in this cohort of patients with NDDs. CNVs smaller than 20 kb were confirmed by quantitative PCR or MLPA.

### Spectrums of Diagnostic SNVs

Of the 423 diagnostic SNVs (pathogenic or likely pathogenic) identified in this study, a total of 267 (63.1%) were found to be novel and involved genes that have been previously reported to cause diseases (eTable 1 in Supplement 2). Among these diagnostic SNVs, 177 (41.8%) were missense variants, 91 (21.5%) were frameshift variants, 91 (21.5%) were stop-gain variants, 47 (11.1%) were splicing variants, 13 (3.1%) were inframe variants, and 4 (0.9%) were synonymous variants (Figure 3). Among the 357 patients diagnosed by SNVs, 228 cases (63.9%) were found to have autosomal dominant heterozygous variants. Additionally, 71 patients (19.9%) were diagnosed with autosomal recessive diseases, with 8 (2.2%) having homozygous variants and 63 (17.6%) having compound heterozygous variants. Furthermore, 58 patients (5.2%) were diagnosed with variants originating from X-linked genes, consisting of 22 hemizygous males (37.9%) and 36 heterozygous females (62.1%). A total of 271 diagnostic variants of the 423 were identified as de novo variants (DNVs), accounting for 64.1% of the total.

## Discussion

In recent years, the simultaneous detection of genomic SNVs, CNVs, and UPD variants has been made possible through the use of ES.^24,25,26,27^ In this study, ES tests were performed for 1106 patients with NDDs and their biologic parents, resulting in an overall diagnostic yield of 46.1% (510 of 1106) through the simultaneous analysis of SNVs, CNVs, and UPD using trio-ES data. Dong et al^18^ conducted an analysis of SNVs and CNVs using clinical ES in a cohort of 1090 patients with developmental disorders and reported an overall diagnostic yield of 41.4%. Subsequently, several studies have been published that reported diagnostic yields of simultaneous SNV and CNV analysis based on ES data in cohorts with developmental disorders or ID ranging from 53.5% to 58.8%.^19,20,28^ These findings suggest that simultaneous analysis of SNVs and CNVs based on ES data could improve the diagnostic yield compared with the average 38% diagnostic yield achieved by only detecting SNVs.^29^ Nonetheless, the results derived from these study cohorts^19,20,28^ are not comprehensive or representative due to the limited sample size, preselected patients, and number of genes contained in the clinical exome. To our knowledge, high-quality studies of consecutive patients with NDDs using trio-ES as the initial genetic testing are not yet available. Therefore, the extensive application of trio-ES and its practicality and cost-effectiveness require validation in large clinical cohorts.^5,19^ To our knowledge, this study represents the most extensive and systematic single-center cohort investigation using trio-ES among children with NDDs. Furthermore, to our knowledge, it is the first study to concurrently provide the genetic spectrums of SNVs, CNVs, and UPD identified in Chinese children with NDDs through the use of trio-ES.

DNVs have been demonstrated to be a major genetic contributor to NDD.^30,31^ Trio-ES has proven to be a rapid and efficient method for uncovering genetic causes in patients with NDD.^31,32,33,34^ Compared with the single-proband approach, trio-ES provides sequence data from parents, enabling precise and immediate identification of DNVs.^32,33,35^ The findings of this study indicate that in patients with NDDs, a significant proportion of cases (401 of 506 [79.2%]) were attributable to DNVs. A total of 141 of the 155 disease-causing CNVs (91.0%) were identified as de novo, while in the 355 patients diagnosed with SNVs, 267 (75.2%) were DNVs. These results are consistent with previous studies that have demonstrated the high proportion of disease-causing DNVs in cohorts with developmental disorders^30,35^ or ID.^33,36^ The overall diagnosis yield of de novo SNVs in this study was as high as 24.1% (267 of 1106), which is comparable to earlier reports that DNVs had 23% potentially pathogenic variants in a cohort with developmental disorders.^32^ Collectively, our results provide further evidence that DNVs may be the most common genetic cause of NDDs in children and that the whole ES test strategy of using proband-parent trios may be effective in detecting DNVs.

Numerous studies have demonstrated that disease-causing CNVs account for approximately 10% to 20% of NDD cases.^17,37,38,39^ Despite the high diagnostic yield of SNVs,^10,29,32,33,35^ ES is limited by nonuniform read depths caused by capture bias in detecting CNVs.^40^ Therefore, CMA remains the preferred genetic testing tool for identifying CNVs in patients with NDDs. However, several tools have been developed for analyzing CNVs based on ES data, such as XHMM,^41^ CODEX,^42^ CANOES,^43^ CoNIFER,^44^ inCNV,^25^ ExomeCNV,^24^ and ExomeDepth.^45^ These tools provide valuable options for enhancing the detection and analysis of CNVs alongside SNVs, potentially optimizing the cost-effectiveness of genetic testing procedures. Dong et al^18^ found a 12% diagnostic yield for CNVs in 1090 patients with developmental disorders using clinical ES data, while Zhai et al^19^ achieved an 18.9% diagnosis yield for CNVs in 74 patients with NDDs using trio-ES data. In the aforementioned studies, the former had limitations owing to a relatively small number of genes and single proband detection, whereas the results of the latter were influenced by small sample sizes and high patient selection bias. Therefore, it is imperative to validate whether CNV analysis based on trio-ES data can replace CMA in clinical genetic testing for patients with NDDs in a larger cohort. In this study, we achieved a diagnostic yield of 13.5% for CNVs, which was based on trio-ES data in a cohort of 1106 children with NDDs and slightly exceeded the average diagnostic yield of 12.2% reported for CMA in previous studies.^38,46^ Our results suggest the equivalence of ES compared with CMA in the detection of CNVs in a cohort with NDDs.

Clinically effective resolution of CMA requires a delicate balance between sensitivity and specificity. Currently available clinical CMA platforms offer a maximum resolution of approximately 20 kb in targeted regions.^3,38^ The detection resolution for CNVs based on ES or clinical ES data are largely dependent on the probe design.^24^ In our study, diagnostic CNVs ranged in size from 124 bp (patient P0957) to 155 Mb (whole chromosome) (patients P0287, P0428, and P1031), notably including 16 small-segment CNVs with sizes less than 20 kb, which are typically undetectable using traditional CMA. Among the patients with these 16 CNVs, 14 (87.5%) were diagnosed with exon-level CNV within a single gene and 2 (12.5%) were codiagnosed with both SNVs and CNVs. The diagnostic yield of these small CNVs was 1.4% (16 of 1106). This increase was often missed in prior clinical genetic testing of patients with NDDs using CMA and ES alone. Of particular interest is patient P0927 (eFigure in Supplement 1), who presented with a single exon deletion that did not overlap with the SNV. In such cases, CNV detection is crucial, as these variants would otherwise be missed.

Our findings suggest that ES data analysis is a more efficient method for CNV detection than traditional CMA. Additionally, UPD has been recognized as a contributing factor to genetic disorders,^47^ known to cause diseases by either homozygous pathogenic variants in recessive genes or absence of imprinted genes. UPD identification can be facilitated through the use of trio-ES data.^24,48^ In the cohort with NDDs in our study, we identified 4 patients with UPD, confirmed by methylation-specific MLPA to cause abnormal methylation, contributing to a diagnostic yield of 0.4% (4 of 1106). This percentage aligns with a previous study that detected CNV using trio-ES data within the Deciphering Developmental Disorders project.^48^ These results suggest that UPD analysis using trio-ES data can enhance the yield of clinical genetic diagnosis without incurring additional testing expenses.

### Limitations

This study has limitations. Although the enrollment criteria for patients in this study were primarily based on clinical phenotypes, the focus was mainly on GDD-ID, ASD, CAs, EIS, ADHD, and abnormal brain MRI findings. The study did not conduct detailed systematic analyses of other phenotypes, such as visual impairment, hearing disorders, and disorders of sex development, nor did it perform quantitative evaluations for patients with ASD or ID. Therefore, in future studies, systematic analysis of patient phenotypes based on different genetic variations should be carried out to provide a basis for clinical treatment and management. Additionally, although this study used trio-ES data to simultaneously analyze CNVs and SNVs, which improved the diagnostic yield of NNDs compared with data analyzing SNVs only, the limitation of ES in detecting genomic noncoding region variants led to some NDD cases of genetic etiology remaining undiagnosed. Another limitation is that ES testing cannot accurately detect NDDs caused by repeat expansions, such as fragile X syndrome and myotonic dystrophy type 1.

## Conclusions

In this cohort study, a retrospective analysis demonstrated that simultaneously detecting SNVs and CNVs in children with NDDs achieved a diagnostic yield of 46.1%, which is equivalent to that obtained using CMA and ES separately.^29,38,46^ Moreover, this approach proved to be advantageous for detecting CNVs smaller than 20 kb and identifying recessive genetic diseases diagnosed jointly by SNVs and CNVs. Our findings suggest that in clinical practice, analyzing CNVs while detecting SNVs with trio-ES data could reduce the reliance on traditional CMA in the genetic diagnosis of children with NDDs while simultaneously identifying SNVs. In the cohort in this study, the remaining undiagnosed patients could potentially benefit from future diagnostic efforts involving reanalysis of ES data,^49^ whole-genome sequencing, and use of multiomics technologies.^50^

## Notes

Supplement 1. **eMethods.** Supplementary Methods **eFigure.** Characterization of Patients Diagnosed With Homozygous or Compound Heterozygous Mutations Involving Single Nucleotide Variants (SNVs) and Copy Number Variants (CNVs)
eReferences

Supplement 2. **eTable 1.** Profiles of Diagnostic SNVs Identified by Trio-ES in Patients With Neurodevelopmental Disorders **eTable 2.** Profiles of CNVs Identified by Trio-ES in Patients With Neurodevelopmental Disorders **eTable 3.** Profiles of UPD Identified by Trio-ES in Patients With Neurodevelopmental Disorders **eTable 4.** Profiles of Variants of Uncertain Significance Identified by Trio-ES in Patients With Neurodevelopmental Disorders

Supplement 3.
Data Sharing Statement
