# Supplementary material for: Diagnostic Utility of Trio–Exome Sequencing for Children With Neurodevelopmental Disorders
Source: JAMA Netw Open. 2025 Mar 25;8(3):e251807. doi: 10.1001/jamanetworkopen.2025.1807 (PMC11937947; doi:10.1001/jamanetworkopen.2025.1807)
Supplement: Supplement 1. — eMethods. Supplementary Methods eFigure. Characterization of Patients Diagnosed With Homozygous or Compound Heterozygous Mutations Involving Single Nucleotide Variants (SNVs) and Copy Number Variants (CNVs) eReferences [file jamanetwopen-e251807-s001.pdf]

Supplementary Online Content

Lan X, Tang X, Weng W, et al. Diagnostic utility of trio-exome sequencing for children with neurodevelopmental disorders. *JAMA Netw Open*. 2025;8(3):e251807.
doi:10.1001/jamanetworkopen.2025.1807

eMethods. Supplementary Methods
eFigure. Characterization of Patients Diagnosed With Homozygous or Compound Heterozygous Mutations Involving Single Nucleotide Variants (SNVs) and Copy Number Variants (CNVs)
eReferences

This supplementary material has been provided by the authors to give readers additional information about their work.

© 2025 Lan X et al. *JAMA Network Open*.

eMethods. Supplementary Methods

Exome Sequencing and Data Analysis

We collected peripheral venous blood (2 ml) from the patients and their parents. Genomic DNA was extracted from the whole blood using the QIAamp Blood Mini Kit (Qiagen, Hilden, Germany). The quality of the DNA was assessed via agarose gel electrophoresis, and its purity and concentration were measured using a NanoDrop™ spectrophotometer (Thermo Fisher Scientific). For next-generation sequencing (NGS), library preparation was conducted using a custom capture probe system based on IDT's xGen Exome Research Panel V2 (Integrated DNA Technologies), following the manufacturer's protocol. The prepared libraries were sequenced on an Illumina NovaSeq 6000 platform (Illumina, San Diego, CA, USA) to produce 150 bp paired-end reads with an average depth of 100x. All samples were aligned to the consensus human genome GRCh37/hg19 using BWA (v 0.7.17).[1] Single nucleotide variant (SNV) calling were performed using the Genome Analysis Toolkit's (GATK 4.4.0.0).[2]

CNV analysis

To determine the copy ratio of each exon, we considered identical conditions for library preparation, capture, and sequencing. This was done by calculating the ratio between the standard coverage of the target exon and the standard coverage of a control group (excluding the target exon itself). Copy number variants (CNV) analysis of exome sequencing (ES) data was conducted using an in-house tool named CNVexon, which relies on coverage and sequencing depth. This custom algorithm is similar to established methods like ExomeCNV and ExomeDepth. It is important to note that a minimum of four samples of the same sex within the same batch is required to serve as controls for the analysis. However, it is common to have a larger number of comparators within the same batch, typically up to 48 samples for ES. The standard score, or z-score, is calculated by dividing the difference between the coverage of the target exon and the control coverage by its standard deviation. The z-score serves as a doubly normalized parameter, effectively mitigating both intra-sample and inter-sample variations. A copy ratio greater than 1.2 and a z-score greater than 1 suggest potential duplications in the target exon, whereas a copy ratio less than 0.8 and a z-score less than -1 indicate potential deletions. Consecutive exons within the same region are independently clustered based on their copy ratio and z-score. Identifying potentially positive CNV clusters requires a combined score of at least ≥ 5 SD from the mean, with at least one exon showing a copy ratio of ≤ 0.6 or ≥ 1.4. This method allows for the reliable detection of deletions/duplications spanning three contiguous exons with a sensitivity greater than 99%. Deletions/duplications involving two consecutive exons can be detected with a sensitivity exceeding 98%, while the sensitivity for detecting those involving a single exon is estimated at 96%. Identified CNVs were interpreted based on their size, frequency, number of key genes, and overlap with known

disease-associated regions, following the standards and guidelines for technical standards for the interpretation and reporting of constitutional copy-number variants: a joint consensus recommendation of the ACMG and the Clinical Genome Resource (ClinGen).[3] The frequencies of CNVs were obtained from reliable sources such as the Database of Genomic Variants (DGV), gnomAD (v2.1.1) and in-house databases. The suspected CNVs smaller than 300 kb were validated by multiplex ligation-dependent probe amplification (MLPA) or real time quantitative PCR (qPCR).

UPD analysis

Uniparental disomy (UPD) can be identified through loss of heterozygosity (LOH) analysis and by determining the origin of homozygous variants using trio-ES data. The LOH analysis was conducted using a custom algorithm that first establishes seed regions consisting of at least 40 consecutive homozygous SNV calls, without any heterozygous SNVs or gaps of 3 Mb between variants. These seed regions are then expanded using a scoring system: the score increases by 0.025 for each additional homozygous variant and decreases significantly, by 0.975, for each heterozygous SNV. Each region is extended at both ends until it reaches the end of a chromosome, encounters a gap of 7.5 Mb between SNVs/indels, or when the score drops to zero. Overlapping regions are merged into a single region. There is no set maximum size for these regions, but they always terminate at the boundary of a chromosome. The origin of homozygous variants within the LOH region is determined through SNV analysis in trio-ES data. Suspected UPDs are subsequently confirmed by methylation-specific multiplex ligation-dependent probe amplification (MS-MLPA), if available.

Variant Interpretation and classification

The annotation and interpretation of SNVs were performed with ANNOVAR software.[4] The variants that had a minor allele frequency higher than 0.05 in the public databases (dbSNP, ExAC, gnomAD v2.1.1, and ESP) and our in-house database were excluded. We employed the SIFT (v6.2.1), PolyPhen_2 (v2.2.3), Mutation Taster (v2021), REVEL, PROVEAN, and CADD (v1.6) to predict pathogenicity or deleteriousness of missense variant. The impact of variants on alternative splicing was predicted by human Splicing Finder[5] and SpliceAI (v1.3.1).[6]The databases such as ClinVar (2024.03), OMIM (2024.03), HGMD (2023.01), and PubMed were consulted to scrutinize variants previously recorded or reported for assessing the pathogenicity. The variants with annotations were classified as pathogenic (P), likely pathogenic (LP), and variants of uncertain significance (VUS) according to the Standards and Guidelines for the Interpretation of Sequence Variants of the American College of Medical Genetics and Genomics (ACMG).[7]

The identified CNVs were interpreted based on their size, frequency, number of key genes, and overlap with

© 2025 Lan X et al. *JAMA Network Open*.

known disease-associated regions, following the standards and guidelines for technical standards for the interpretation and reporting of constitutional copy-number variants: a joint consensus recommendation of the ACMG and the Clinical Genome Resource (ClinGen).[3] The frequencies of CNVs were obtained from reliable sources such as the Database of Genomic Variants,[8] gnomAD and in-house database. The suspected CNVs smaller than 100 kb were validated by multiplex ligation-dependent probe amplification (MLPA) or quantitative PCR (qPCR). The suspected UPDs were subsequently confirmed by methylation-specific MLPA (MS-MLPA) if available.

© 2025 Lan X et al. *JAMA Network Open*.

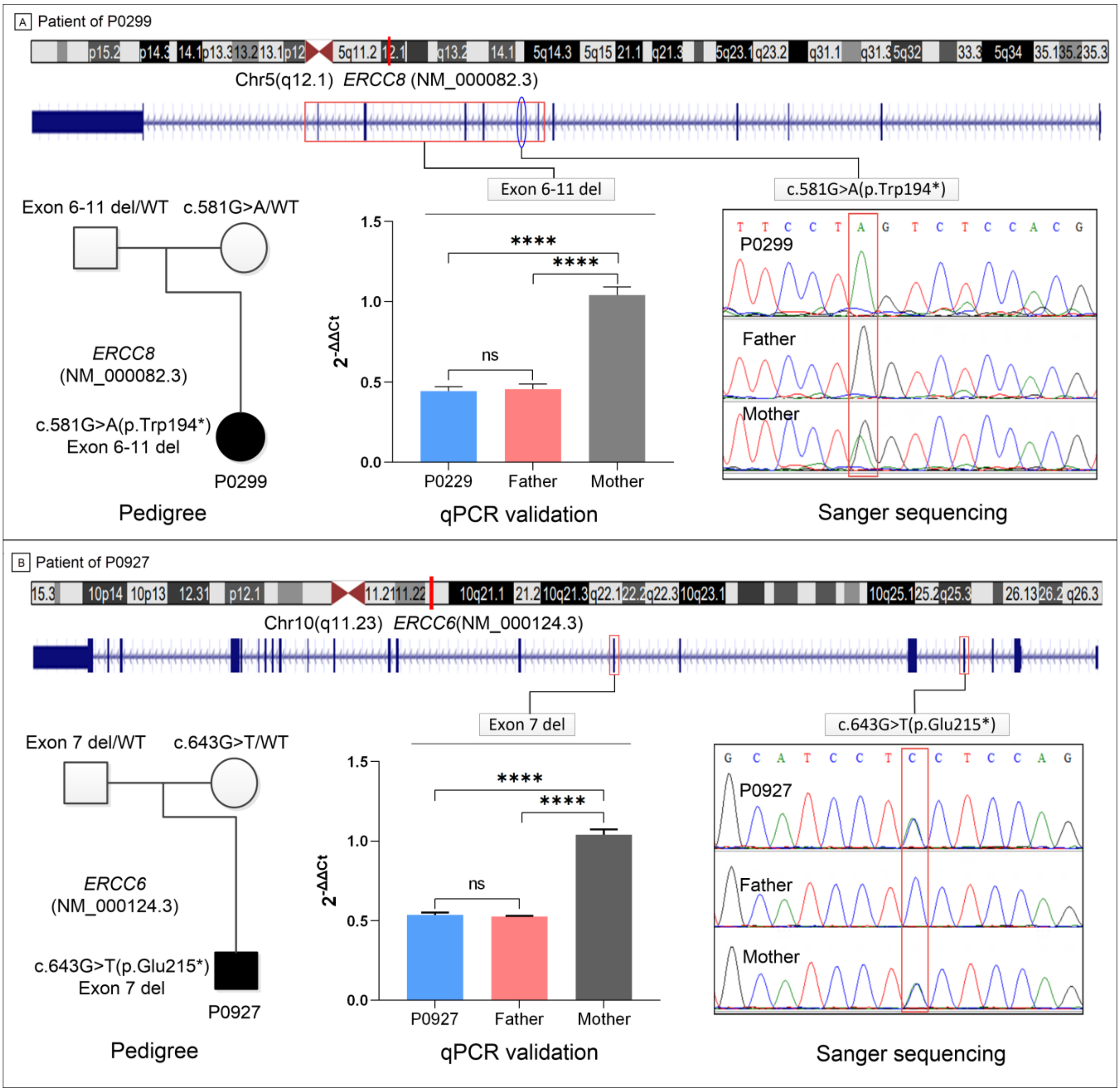

eFigure . Characterization of patients diagnosed with homozygous or compound heterozygous mutations involving single nucleotide variants (SNVs) and copy number variants (CNVs). (A) Patient P0299. (B) Patient P0927.

© 2025 Lan X et al. *JAMA Network Open*.

eReferences

1. Li H, Durbin R. Fast and accurate short read alignment with Burrows-Wheeler transform. *Bioinformatics.* 2009;25(14):1754-1760.
2. Van der Auwera GA, Carneiro MO, Hartl C, et al. From FastQ data to high confidence variant calls: the Genome Analysis Toolkit best practices pipeline. *Current protocols in bioinformatics.* 2013;43(1110):11 10 11-11 10 33.
3. Riggs ER, Andersen EF, Cherry AM, et al. Technical standards for the interpretation and reporting of constitutional copy-number variants: a joint consensus recommendation of the American College of Medical Genetics and Genomics (ACMG) and the Clinical Genome Resource (ClinGen). *Genet Med.* 2020;22(2):245-257.
4. Yang H, Wang K. Genomic variant annotation and prioritization with ANNOVAR and wANNOVAR. *Nat Protoc.* 2015;10(10):1556-1566.
5. Desmet FO, Hamroun D, Lalande M, Collod-Beroud G, Claustres M, Beroud C. Human Splicing Finder: an online bioinformatics tool to predict splicing signals. *Nucleic Acids Res.* 2009;37(9):e67.
6. Jaganathan K, Kyriazopoulou Panagiotopoulou S, McRae JF, et al. Predicting Splicing from Primary Sequence with Deep Learning. *Cell.* 2019;176(3):535-548 e524.
7. Richards S, Aziz N, Bale S, et al. Standards and guidelines for the interpretation of sequence variants: a joint consensus recommendation of the American College of Medical Genetics and Genomics and the Association for Molecular Pathology. *Genet Med.* 2015;17(5):405-424.
8. MacDonald JR, Ziman R, Yuen RK, Feuk L, Scherer SW. The Database of Genomic Variants: a curated collection of structural variation in the human genome. *Nucleic Acids Res.* 2014;42(Database issue):D986-992.

© 2025 Lan X et al. *JAMA Network Open*.
