# Supplementary material for: Diagnostic Utility of Trio–Exome Sequencing for Children With Neurodevelopmental Disorders
Source: JAMA Netw Open. 2025 Mar 25;8(3):e251807. doi: 10.1001/jamanetworkopen.2025.1807 (PMC11937947; doi:10.1001/jamanetworkopen.2025.1807)
Supplement: Supplement 3. — Data Sharing Statement [file jamanetwopen-e251807-s003.pdf]

Data Sharing Statement

Lan. Diagnostic Utility of Trio–Exome Sequencing for Children With Neurodevelopmental Disorders. *JAMA Netw Open*. Published March 25, 2025.
doi:10.1001/jamanetworkopen.2025.1807

Data
Data available: No

Additional Information
Explanation for why data not available: Individual-level data are not publicly available due to ethical and legal restrictions related to the Shanghai Children's Hospital.
